# Quantitative T2 Combined with Texture Analysis of Nuclear Magnetic Resonance Images Identify Different Degrees of Muscle Involvement in Three Mouse Models of Muscle Dystrophy: *mdx, Large^myd^* and *mdx/Large^myd^*

**DOI:** 10.1371/journal.pone.0117835

**Published:** 2015-02-24

**Authors:** Aurea B. Martins-Bach, Jackeline Malheiros, Béatrice Matot, Poliana C. M. Martins, Camila F. Almeida, Waldir Caldeira, Alberto F. Ribeiro, Paulo Loureiro de Sousa, Noura Azzabou, Alberto Tannús, Pierre G. Carlier, Mariz Vainzof

**Affiliations:** 1 Centro de Estudos do Genoma Humano, Departamento de Genética e Biologia Evolutiva, Instituto de Biociências, Universidade de São Paulo—IB-USP, São Paulo, São Paulo, Brazil; 2 Centro de Imagens e Espectroscopia in vivo por Ressonância Magnética—CIERMag. Instituto de Física de São Carlos, Universidade de São Paulo—IFSC-USP, São Paulo, São Paulo, Brazil; 3 Departamento de Fisiologia, Universidade Federal de São Paulo—UNIFESP, São Paulo, São Paulo, Brazil; 4 Laboratório de Microscopia Eletrônica, Departamento de Genética e Biologia Evolutiva, Instituto de Biociências, Universidade de São Paulo—IB-USP, São Paulo, São Paulo, Brazil; 5 Université de Strasbourg, CNRS, ICube (UMR 7357), FMTS, Strasbourg, France; 6 NMR Laboratory, Institute of Myology, Paris, France; 7 CEA, I²BM, MIRCen, NMR Laboratory, Paris, France; Institut de Myologie, FRANCE

## Abstract

Quantitative nuclear magnetic resonance imaging (MRI) has been considered a promising non-invasive tool for monitoring therapeutic essays in small size mouse models of muscular dystrophies. Here, we combined MRI (anatomical images and transverse relaxation time constant—T2—measurements) to texture analyses in the study of four mouse strains covering a wide range of dystrophic phenotypes. Two still unexplored mouse models of muscular dystrophies were analyzed: The severely affected *Large^myd^* mouse and the recently generated and worst double mutant *mdx/Large^myd^* mouse, as compared to the mildly affected *mdx* and normal mice. The results were compared to histopathological findings. MRI showed increased intermuscular fat and higher muscle T2 in the three dystrophic mouse models when compared to the wild-type mice (T2: *mdx/Large^myd^*: 37.6±2.8 ms; *mdx*: 35.2±4.5 ms; *Large^myd^*: 36.6±4.0 ms; wild-type: 29.1±1.8 ms, p<0.05), in addition to higher muscle T2 in the *mdx/Large^myd^* mice when compared to *mdx* (p<0.05). The areas with increased muscle T2 in the MRI correlated spatially with the identified histopathological alterations such as necrosis, inflammation, degeneration and regeneration foci. Nevertheless, muscle T2 values were not correlated with the severity of the phenotype in the 3 dystrophic mouse strains, since the severely affected *Large^myd^* showed similar values than both the mild *mdx* and worst *mdx/Large^myd^* lineages. On the other hand, all studied mouse strains could be unambiguously identified with texture analysis, which reflected the observed differences in the distribution of signals in muscle MRI. Thus, combined T2 intensity maps and texture analysis is a powerful approach for the characterization and differentiation of dystrophic muscles with diverse genotypes and phenotypes. These new findings provide important noninvasive tools in the evaluation of the efficacy of new therapies, and most importantly, can be directly applied in human translational research.

## Introduction

The muscular dystrophies are an extensive group of genetic diseases where the major characteristic is the progressive muscle degeneration, caused by mutations in genes coding for sarcolemmal, sarcomeric, cytosolic, nuclear or extracellular matrix proteins. The absence or altered function of one of these proteins is responsible for a cascade of events which ends in the muscle fibers degeneration and substitution by connective and adipose tissue. The patients present progressive weakness, starting at different ages depending on the mutation. Up to now, there is no effective cure for this group of diseases, and several therapeutic protocols are in development [1, 2].

The most frequent form of muscular dystrophy is Duchenne Muscular Dystrophy (DMD), caused by mutations in the dystrophin gene and with an incidence of 1 in 3300 live male births [3, 4]. The dystrophin protein is part of the dystrophin-glycoprotein complex (DCG), which links the cytoskeleton from muscle fibers to the extracellular matrix. This connection is mediated by the dystroglycan complex, composed by the sarcolemmal beta-dystroglycan (β-DG) subunit and the peripheral membrane alpha-dystroglycan (α-DG). While β-DG links to the subsarcolemmal protein dystrophin, α-DG is responsible for the connection with the extracellular matrix protein α-2 laminin. This link occurs via the sugar chains in the glycosylated extension of α-DG, which have high affinity to Laminin G (LG)-like domains present in various extracellular matrix proteins, such as laminins, perlecan and agrin in muscle, and neurexin in brain [5, 6, 7]. Mutations in the gene coding for dystroglycans are very rare, but alterations in α-DG glycosylation are related to several forms of myopathy, such as limb girdle muscular dystrophies and congenital muscular dystrophies [8].

The study of animal models for neuromuscular disorders has an essential role in understanding the pathogenetic mechanisms of the muscular diseases and in the development of therapeutic strategies. There are several natural or created animal models for the different forms of muscle dystrophy, which can model the genetic, molecular and/or clinical aspects of the disease. The *Dmd* ^*mdx*^ mouse (hereafter called simply *mdx*) is the most frequently used mouse model for DMD. This mouse has a stop codon in exon 23 of the murine dystrophin gene, which leads to the total absence of this protein in the muscle, as observed in DMD patients [3, 9, 10]. Nevertheless, differently from the human patients, the *mdx* mouse can continuously regenerate its muscles and has a mild phenotype, which makes the analysis of functional benefices in therapeutic protocols very difficult [11, 12].

Double mutant mice with the *mdx* background have been created in the attempt to approach the severe phenotype observed in DMD patients, such as the double knockout *mdx*:*utrn* ^*-/-*^, with absence of both dystrophin and utrophin [13, 14]; the *mdx/mTR* mouse, with impaired telomerase activity [15]; and the *Dmd* ^*mdx*^ *-Large* ^*myd*^ mouse (hereafter called *mdx/Large* ^*myd*^), recently generated in our laboratory by crossing *mdx* and *Large* ^*myd*^ murine lineages [16]. The *Large* ^*myd*^ myodystrophy mouse has a mutation in the glycosyltransferase Large gene, which leads to reduced glycosylation of α-DG and a severe and progressive myodystrophy. Mutations in the human gene *LARGE* are related to congenital muscular dystrophy 1D (CMD1D), with severe muscle and central nervous system involvement. The double mutant *mdx/Large* ^*myd*^ mouse presents deficiency of both dystrophin and Large proteins, and a very severe phenotype, worse than both parental lineages. The lifespan is reduced and the degree of muscle degeneration and infiltration by connective tissue is increased when compared to the parental lineages. The *mdx/Large* ^*myd*^ mouse gives clues of the interplay between α-DG glycosylation and dystrophin deficiency and is useful for testing therapies due to the functional, genetic and protein alterations [16].

Different therapeutic strategies for muscular dystrophies are in development, including genetic and cellular approaches. The gold standard to evaluate the dystrophic muscle is still the histological analysis, but non-invasive methods are highly desirable. Nuclear magnetic resonance (NMR), and more specifically magnetic resonance imaging (MRI), have a great potential in the study of skeletal muscle due to its flexibility in generating images from soft tissues with different contrasts, additionally enabling metabolic and functional studies. MRI studies in dystrophic patients have revealed different patterns of muscle involvement depending on the mutation, which can be used to orient the molecular testing in the differential diagnosis [17, 18]. Human DMD clinical evolution and muscle metabolism alterations can be non-invasively tracked by NMR, with a good correlation between quantitative NMR parameters and the clinical evaluation [19, 20, 21, 22, 23, 24, 25, 26].

When applied to small animals, MRI faces the challenge of reducing dimensions and increasing resolution. Additionally, differently from human patients, mouse models of muscular dystrophies present low to zero fat infiltration in the muscle [27, 28]. The MRI analysis of intra-muscular fat infiltration is therefore not as informative in mice as it is in patients, which prompted the search for other MRI approaches to non-invasively evaluate mouse models, such as transverse relaxation time constant (T2) measurements and muscle texture analysis.

T2 is an NMR value intrinsic for each type of tissue, reflecting the motility of its water protons. When the examined tissue presents pathologic processes such as necrosis, inflammation or edema, this dynamic is altered, which results in changes in the tissue T2. Previous studies have reported abnormal muscle T2 in some dystrophic models, such as *mdx* [28, 29, 30], γ-sarcoglycan knock-out γsg^-/-^ [29], α-sarcoglycan knock-out sgca^-/-^ [31], and α-2 laminin deficient *dy/dy and dy* ^*2J*^ */dy* ^*2J*^ mouse models [32], as compared to normal controls. Texture analysis is an emerging approach that includes several techniques to quantify variations in the image intensity or patterns. When applied to muscle MRI, texture analysis has demonstrated to be a potential tool to evaluate subtle differences in the pattern of distribution of muscle lesions [33, 34, 35]. In the *mdx* mouse [36] and the GRMD dystrophic dog [37], longitudinal studies were able to correlate texture parameters with age and progression of the disease.

Considering this, we hypothesized that T2 measurements combined to muscle MRI texture analysis would be sensitive enough to characterize and differentiate dystrophic muscle phenotypes caused by different gene mutations. Here, we used this approach to evaluate two still unexplored mouse models of muscular dystrophies, the recently generated *mdx/Large* ^*myd*^ mouse and the *Large* ^*myd*^ parental lineage, in addition to *mdx* and C57Bl mice. These mouse strains cover a wide range of dystrophic phenotypes, with variable degrees of muscle necrosis and inflammation, and could be unambiguously identified with quantitative muscle T2 and texture analysis. The new findings will have important applications to noninvasive follow of potential therapeutic protocols.

## Material and Methods

### Ethics Statement

All the experiments were approved by the Research Ethics Committee of the Biosciences Institute, University of São Paulo, protocol 176/2013.

### Animals

Four mouse strains were evaluated: the dystrophic *mdx, Large* ^*myd*^ and *mdx/Large* ^*myd*^, in addition to C57Bl/6 mice as normal controls (wild-type). 47 mice, aged between 2 and 4 months (8–19 weeks), both male and female, were studied: 9 double mutants *mdx/Large* ^*myd*^, 13 *mdx*, 12 *Large* ^*myd*^ and 13 wild-type. As the two models with a weaker phenotype, in particular *mdx/Large* ^*myd*^ mice, are difficult to obtain (25% of affected sibling and high degree of perinatal death, [16]), a more flexible range of ages was adopted. To homogenize the groups, the accepted age range was allowed to vary between 8 and 18 weeks for all mouse strains. At this stage, muscle dystrophy is patent in all mouse strains. This age range is posterior to the critical period in *mdx* mice, when a peak of myofiber necrosis, muscle weakness and regeneration is observed between the 2^nd^ and the 5^th^ weeks of life [38]. All the animals were from the Human Genome Research Center animal house, Bioscience Institute (Sao Paulo). The mice were kept in controlled environment, with water and food at libidum.

### Magnetic Resonance Imaging acquisition and analysis

The mice were anesthetized with intraperitoneal injection of ketamine:xylazine (2:1, 1.5–2.5 μl/g according to the lineage) and symmetrically positioned for the MRI acquisitions. The images were acquired in a 2 tesla/30 cm bore superconducting magnet (Oxford Instruments 85310HR, Abingdon, United Kingdom), interfaced to a Bruker Avance AVIII console (Bruker-Biospin, Inc., Billerica, MA, U.S.A.) running PARAVISION 5.0. A crossed-saddle radiofrequency coil projected for small animals [39] was used to image the mice's posterior limbs.

Four scans were performed in each mouse, with the same geometry (4 slices, 1.5 mm slice thickness, 4 mm inter-slice distance and spatial resolution 0.176X0.176 mm^2^/pixel): two scans for anatomical images with the same parameters (repetition time-TR = 1800 ms, echo time - TE = 52.5 ms), with and without fat suppression for qualitative evaluation of possible fat infiltration in the muscles (with fat suppression:16 averages; without fat suppression: 4 averages); and two scans for the calculation of the T2 maps, each one using a different echo time (to avoid the contribution of stimulated echoes): TE_1_ = 12.1 ms and TE_2_ = 40 ms (TR = 1500 ms, 1 average when TE_1_ = 12.1 ms, 4 averages when TE_2_ = 40 ms, spatial resolution: 0.176X0.176 mm^2^/pixel). The T2 value for each pixel was calculated using the Bloch equation for the spin-spin relaxation time (S1 Supporting Information), and T2 maps were generated using a routine developed in the MATLAB software (The MathWorks, Inc., Natick, Massachusetts, USA). The total acquisition time was 51 minutes. The examination time never exceeded 1 hour and 30 minutes.

In the T2 maps, two slices were selected for analysis: one positioned at the lower leg and one at the thigh. One slice was considered representative of thigh and leg muscles since injured fibers would present anomalies along all its length. Nevertheless, this limits the analysis to the muscles observed in a defined anatomical position.

Four Regions of Interest (ROI) were evaluated: two in the lower leg T2 map, at the posterior and anterior compartment muscles, and two in the thigh T2 maps, covering the medial and lateral muscles. The ROIs were drawn to exclude any non-muscle tissue. For each ROI, the mean T2 value and the standard deviation were analyzed.

The muscle texture in MRI was evaluated with the softwares MaZda 4.6 and B11 3.3 [40, 41, 42], using the T2-weighted images acquired with TE = 40 ms and TR = 1500 ms. The lower leg image was selected and one ROI was drawn in the left lower leg of each mouse, including all muscle groups but excluding subcutaneous fat, bones and skin. All the 371 texture features offered by the Mazda software were calculated. The co-occurrence matrices parameters contrast and entropy were selected due to their significance for the identification of our studied groups, and resulted in the reduction of the 371 features to 40. Among them, Mazda software automatically selected 30 features, by combining the maximization of the Fischer coefficients, the maximization of the mutual information between two selected features, the minimization of the classification error probability and minimization of the average correlation coefficients (F+PA+MI). The 30 selected features (listed in S2 Supporting Information) were then used as input to Linear Discriminant Analysis (LDA) in the software B11.

### Histological analysis

Two days after the NMR session the mice were euthanized in a CO_2_ chamber. The whole left leg was collected and preserved in formaldehyde solution (4% in Phosphate Buffer Saline - PBS). The right leg was dissected and the lower leg posterior compartment (gastrocnemius and soleus) was imbedded in Tissue-tek OCT freezing medium (Optimal Cutting Temperature, Sakura Finetek USA, Torrance, CA, USA) and frozen in liquid nitrogen.

The histological qualitative evaluation was performed on at least 2 mice for each lineage. The formaldehyde preserved samples were decalcified and embedded in plastic resin according to the manufacturer procedure (HistoResin, Leica, Wetzlar, Germany). Resin embedded samples were cut in 5 μm slices and frozen samples were cut in 8 μm slices. Both frozen and resin embedded histological sections were stained with Hematoxilin and Eosin (H&E). Additional Gomori trichrome and sirius red staining were done in the frozen sections.

### Statistic analysis

The T2 mean values for each ROI were analyzed with a three-factors ANOVA, followed by the Bonferroni muticomparison test when ANOVA detected differences (p<0.05). The three considered factors were: presence of the mutation in the dystrophin gene (*Dmd* ^*+/+*^ = no mutation/Dmd^*-/-*^ = with mutation); presence of the mutation in the gene *Large* (*Large* ^*+/+*^ = no mutation/*Large* ^*-/-*^ = with mutation); and muscle group (1: lower leg posterior, 2: lower leg anterior, 3: thigh lateral, 4: thigh medial). The lineage was split in two factors to evaluate a possible cumulative effect of both mutations in the double mutant *mdx/Large* ^*myd*^ mouse, and the muscle groups were studied separately to evaluate a possible different pattern of muscle involvement in the dystrophic mouse strains. The differences were considered significant when p<0.05. This level of significance was achieved in ANOVA when F(1,164)>3.90 for the comparison of mice from different genetic background and when F(3,164)>2.66 for the comparison of different muscle groups. Analyses were performed with NCSS 2001 software (Kaysville, Utah, USA).

## Results

**Fig 1:**
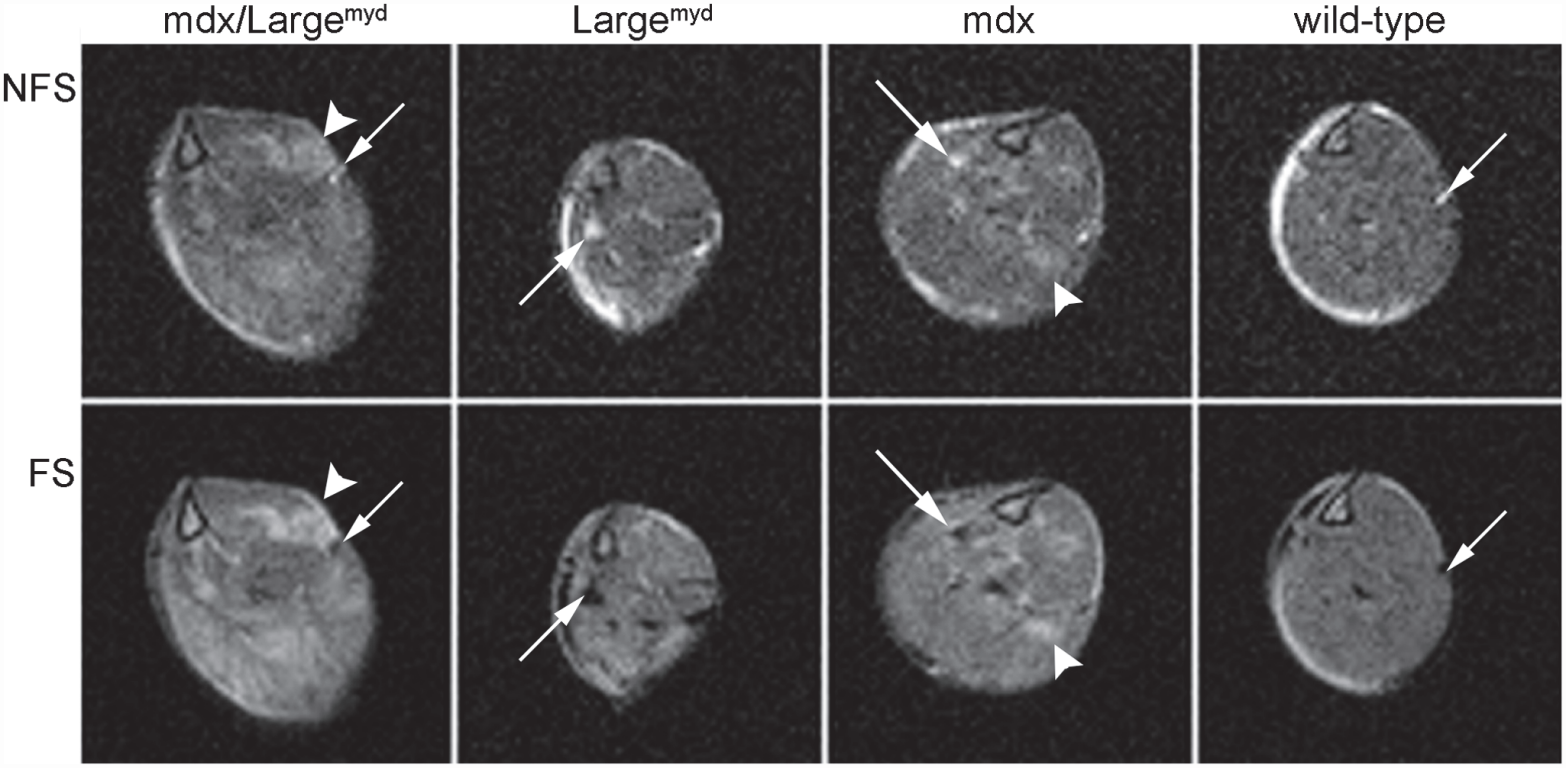
Intermuscular fat in dystrophic mice. MRI of the four mouse strains (lower leg), showing intermuscular fat but no visible fat infiltration in the muscles in the three dystrophic strains. The arrows indicate the presence of fat between the muscles, as bright areas in the non fat-supressed images (NFS) and dark areas in the images with fat suppression (FS). The arrowheads indicate hyperintense areas present in the images with and without FS, which are therefore not related to fat infiltration. TE = 52.5 ms, TR = 1800 ms.

The three dystrophic mouse strains showed increased amount of fat between the muscles when compared to the wild-type mice (Fig. 1). However, no intra-muscular fat could be detected in any of the mouse strains with MRI: the muscle hyperintensities present in the images without fat suppression (Fig. 1, *NFS*) were identically observed in the images with fat suppression (Fig. 1, *FS)*, indicating that these areas were not related to fat infiltration.

### Muscle T2

In the individual comparison between muscle groups for each lineage, only the wild-type mice showed differences: thigh medial muscles have higher muscle T2 than the lower leg muscles (p<0.01). In the three dystrophic strains no differences were observed between the muscle groups. Therefore, all muscles were grouped in one unique T2 value for each animal for the comparison between the mouse strains (Table 1).

**Table 1:** T2 per muscle group in milliseconds for the four mouse strains studied.

	*mdx/Large* ^*myd*^	*mdx*	*Large* ^*myd*^	*wild-type*
N	9	13	12	13
Lower leg - posterior	36.96 ± 2.84	34.95 ± 3.70	35.48 ± 2.15	28.34 ± 1.71
Lower leg - anterior	37.15 ± 3.77	33.43 ± 2.85	33.95 ± 3.59	28.05 ± 1.28
Thigh - lateral	37.70 ± 3.07	36.41 ± 7.20	37.79 ± 5.38	29.69 ± 1.36
Thigh - medial	38.34 ± 1.66	36.31 ± 3.00	38.48 ± 2.54	30.36 ± 1.93
Comparison between muscles	p = 0.76	p = 0.32	p = 0.025	p<0.01
All muscles	37.56 ± 2.82	35.23 ± 4.53	36.58 ± 4.00	29.11 ± 1.82

No differences were observed between the muscle groups in the three dystrophic mouse strains. p-values are for the comparisons between muscle groups for each mouse strain. Since four individual comparisons were done, the Bonferroni correction was applied and the differences were considered significant if p<0.0125.

**Fig 2:**
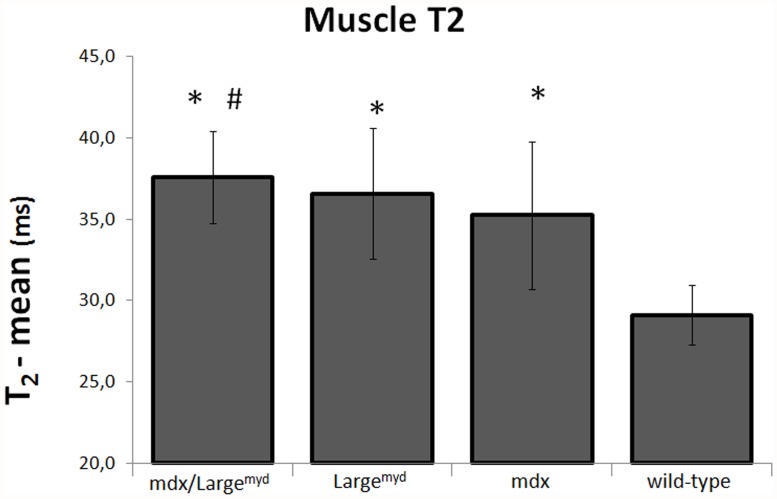
Muscle T2 for *mdx/Large^myd^, Large^myd^, mdx* and wild-type mice. Muscle T2 in milliseconds for the four mouse strains evaluated. *: Muscle T2 different from wild-type mice; #: muscle T2 different from *mdx* mice.

Considering the mouse strains individually, muscle T2 was markedly increased in the three dystrophic mouse models when compared to the wild-type mice (p<0.05). Additionally, *mdx/Large^myd^* mice had significantly higher muscle T2 than *mdx* mice (p<0.05, Table 1, Fig. 2).

**Fig 3:**
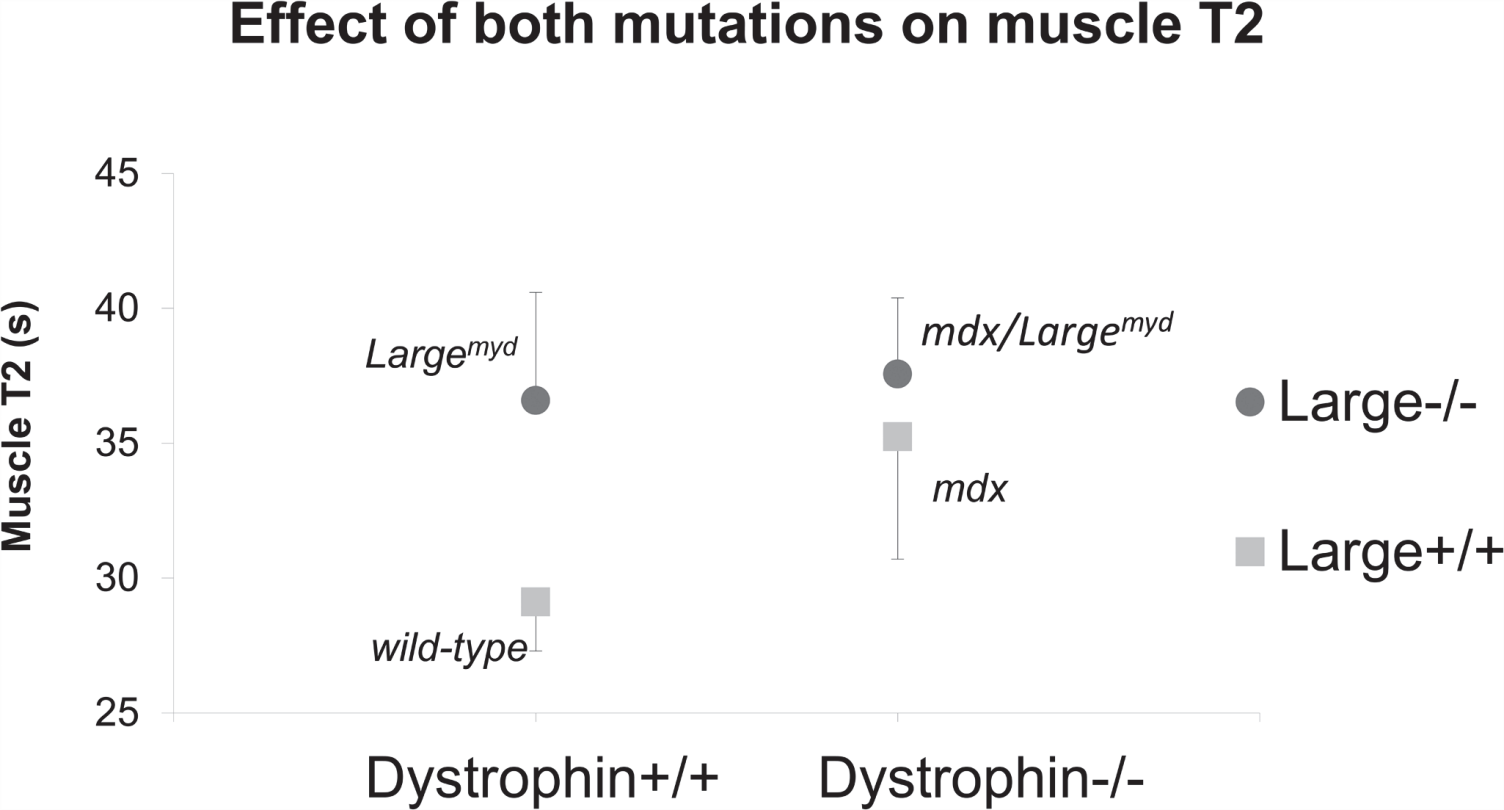
Effect of each mutation on muscle T2. Both the dystrophin absence and the α-DG glycosylation defect increase muscle T2, but the combination of both does not produce an additive effect.

When considering the effect of each gene mutation separately in muscle T2 values, ANOVA showed that there was a significant interaction between the absence of dystrophin and the defective glycosylation of α-DG (p<0.001). The presence of both mutations lead to an increase in muscle T2 when compared to the *mdx* mice, but this increase was lower than the simple sum of the effects of each mutation (Fig. 3).

### Muscle texture analysis

**Fig 4:**
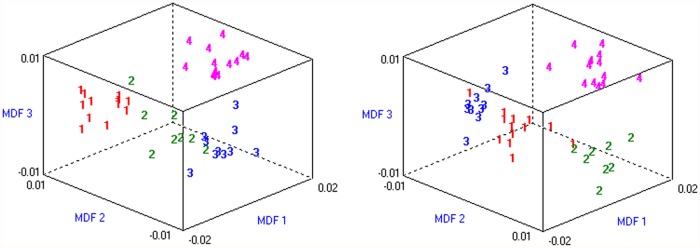
Texture analysis differentiates the four mouse strains. Two views of the same plot showing the clustering of mice groups after texture analysis from lower leg MRI. 1: wild-type, 2: *mdx/Large^myd^*, 3: *Large^myd^*, 4: *mdx* mice. MDF: Most Discriminant Features.

Despite similar values of T2, it was possible to observe differences in the distribution of hyperintense regions in muscle MRI: while *mdx* mice presented focal areas with hyperintense signal randomly distributed in the muscles (patchy), *Large* ^*myd*^ mice presented a global increase in muscle signal, distributed homogeneously in all muscles (waxy). The double mutant *mdx/Large* ^*myd*^ presented a waxy aspect in muscle images, with a few focal hyperintense areas, as a mixture of the patterns observed in the parental strains. The differences in global appearance observed visually in the muscle images from the dystrophic mice were then quantified using texture analysis algorithms. The four groups were properly distinguished with the constructed model, and all the individuals were correctly classified by it. The linear separability was 0.91 and 3 dimensions could model 97% of the original data. Plotting the data over the three dimensions of the model nicely visualized the clustering of the mice into four groups according to the mutation (Fig. 4).

### Histological analysis

**Fig 5:**
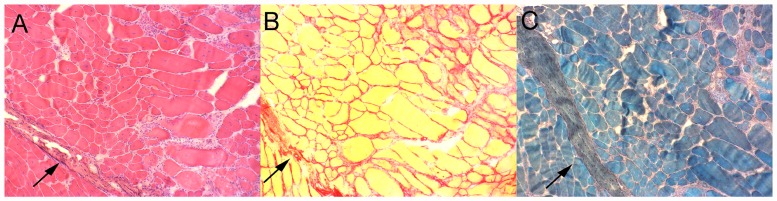
Identification of dystrophic pathological structures. Histological sections of a 3 month-old *mdx/Large* ^*myd*^ mouse (frozen sample), approximately at the same position, stained with (A) Hematoxilin-Eosin, (B) Sirius Red, and (C) Gomori trichrome, magnification X 100. The arrows indicate thick areas of connective tissue.

The histological analysis was based on qualitative comparison of dystrophic pathologic features, such as the presence of degenerating and regenerating cells foci, necrosis, and infiltration by connective and adipose tissues. Homogeneous and pale eosinophilic sarcoplasm was related to degenerating and necrotic fiber, while basophilic sarcoplasm and central nuclei were related to regenerating fibers (Fig. 5) [43, 44].

**Fig 6:**
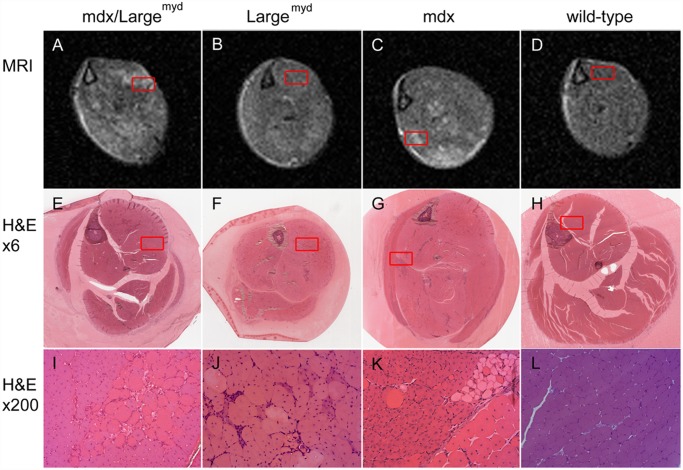
MRI versus histological analysis. MRI (A-D; TE = 40 ms, TR = 1500 ms) and histological images (H&E, magnification X12: E-H; magnification X200: I-L) of the left lower leg from *mdx/Large* ^*myd*^ (A, E, I), Large^myd^ (B, F, J), *mdx* (C, G, K) and wild-type mice (D, H, L). The regions highlighted in the MRI (first row) and in the whole lower leg histological image (second row) are presented in a higher magnification in the third row. Different histological processes could be related to the hyperintensities regions in the MRI, such as clusters of degenerating cells (I), regenerating and adipose cells cells (K), and regions with mixed dystrophic characteristics (J).

Histological sections from the resin embedded samples, which preserved the anatomical organization of the muscle groups, where chosen to represent the MRI slices (Fig. 6).

The *mdx* mice showed a patchy pattern in the MRI (Fig. 6C), and the hyper-signal areas in MRI corresponded spatially to foci of dystrophic alterations, such as clusters of degenerating and regenerating cells, infiltration by inflammatory cells and spots of adipose tissue in the periphery of the muscles (Fig. 6G, K). It was also possible to observe variation in the fibers caliber, high proportion of centronucleated muscle fibers and increased amount of connective tissue in all the lower leg muscles, but these alterations did not necessarily correlate with hyperintense areas in the MRI.

The *Large* ^*myd*^ mice presented dystrophic histological characteristics distributed in a more diffuse pattern through the muscles, in accordance with the waxy pattern observed in the MRI (Fig. 6B, F). The histological analysis revealed the presence of degenerating and regenerating muscle cells, inflammatory infiltrates and fibrosis in a diffuse distribution, with occasional small clusters of degenerating and regenerating fibers. The muscles were generally more compromised than in the *mdx* mice (Fig. 6J).

The *mdx/Large* ^*myd*^ mice showed infiltration by inflammatory cells, fibrosis and degenerating fibers diffusely distributed across the muscles, such as the *Large* ^*myd*^ mice, especially in the posterior compartment muscles of the leg. Additionally, there were few big clusters of degenerating and regenerating fibers which co-localize with hyper-signal areas in the MRI, but in a less accentuated proportion than in the *mdx* mice (Fig. 6A, E). In general, the *mdx/Large* ^*myd*^ mice presented a more severe degree of dystrophic lesions in the histological analysis than both parental mouse strains (Fig. 6I).

The wild-type mice showed polygonal fibers with regular size and peripheral nuclei. There was no fat accumulation inside the muscles, and between the muscles the amount of fat was reduced in comparison to the three dystrophic mouse strains. The endomysial and perimysial connective tissue were thinner than in the three dystrophic mouse strain (Fig. 6H, L). The normal histological pattern was compatible with the homogeneous and low intensity signal in muscle MRI (Fig. 6D).

## Discussion

Noninvasive tools for muscle evaluation have an immediate application in therapeutic protocols, to follow possible benefits in longitudinal studies with minimal impact on the subjects. For this purpose, it is essential that the selected tool could not only detect differences between dystrophic and normal muscle, but that it could also detect more subtle differences between various degrees of muscle involvement, both in patients and in animal models. In this MRI study, we have evaluated for the first time the muscle MRI pattern in the *Large* ^*myd*^ and the double mutant *mdx/Large* ^*myd*^ mouse models of muscular dystrophy, as compared to *mdx* and C57Bl/wild-type mice. These mouse strains cover a wide range of dystrophic phenotypes, from the mildly affected *mdx* mouse to the severely compromised *mdx/Large* ^*myd*^ mouse. Using a combined approach including qualitative MRI evaluation, muscle T2 relaxometry and texture analysis, in comparison to the standard histological analysis, we have shown that noninvasive MRI can successfully discriminate muscles from these four mouse strains.

MRI acquired with and without fat saturation revealed no identifiable fat infiltration in the muscles of any of the mouse strains, but an increased fat accumulation between the muscles in the MRI could be observed in the dystrophic mice when compared to the wild-type. This observation was confirmed by the histological analysis, where it was possible to identify bigger fat deposits between the muscles in the dystrophic mouse strains. Our results extend to the more severely dystrophic *Large* ^*myd*^ and *mdx/Large* ^*myd*^ models the previously described MRI findings in the *mdx* mouse, of no fat infiltration in skeletal muscles visible with MRI [28, 29, 36].

In addition to the increased intermuscular fat, the histological analysis revealed sporadic adipose cells in the muscles of the three dystrophic mouse strains, notably in the *mdx* mice, where small clusters of adipose cells were present especially along the fascia. This is in accordance with previous MRI observation in DMD patients, where increased fat signal along the fascia in young DMD boys was present even when the intramuscular fat replacement was minimal [45]. Although no fat infiltration could be observed in the mice MRI, we believe that the presence of only sporadic and isolated adipose cells in the muscle would not be visually detected in the MRI in the resolution used in this study. It is possible though that this punctual presence of fat in the muscle can lead to changes in the muscle T1 and T2 measurements, in addition to alterations in the MRI muscle texture.

The quantitative evaluation of the mean muscles T2 showed increased values for the three dystrophic mouse strains when compared to the wild-type mice. An increased but not cumulative effect of the dystrophin absence and the α-DG glycosylation defect was observed in the double mutant *mdx/Large* ^*myd*^ mouse: its muscle T2 was higher than the value observed in the *mdx* mouse, but it was not different from the *Large* ^*myd*^ mouse. The increased muscle T2 in the dystrophic mice is in accordance with previous studies showing higher muscle T2 in the *mdx* mouse [28, 29, 30], and in other mouse models of muscular dystrophies such as laminin-deficient mice [32] and the γ-sarcoglycan-null mice [29]. Our NMR study in *Large* ^*myd*^ and *mdx/Large* ^*myd*^ mice extend the number of dystrophic mouse models where increased muscle T2 is observed, corroborating the hypothesis that even if no significant fat infiltration is observed in the MRI of murine models of muscle dystrophy, increased muscle T2 would be a common feature of the dystrophic muscle, both in patients and animal models with variable phenotype.

Muscle water T2 reflects the mobility of water molecules in the tissue. The loss of muscle proteins, the presence of infiltrated adipose and inflammatory cells, and the edema originated by inflammation and necrosis in the dystrophic muscle lead to alterations in the mobility of water molecules, and consequently can contribute to the T2 alterations [46]. The absence of the dystrophin protein destabilizes the DGC and leads to increased membrane damage in the *mdx* mouse. Sarcolemma disruption and increased cell permeability cause edema and extravasation of the sarcoplasmatic content [47], with consequent altered mobility of water molecules and increased T2. Even if the *mdx* mouse has a mild phenotype, the membrane disruptions are present in higher proportion when compared to the more severely affected α-2 laminin-deficient mice, *dy/dy* and *dy* ^*2J*^ */dy* ^*2J*^ [46]. The same pathological disruption of the sarcolemma is observed in cardiac muscle cells from *Large* ^*myd*^ mice [48], and is possibly present also in the skeletal muscles from *Large* ^*myd*^ and the double mutant *mdx/Large* ^*myd*^ mice. Thus, sarcolemma disruption, in addition to the consequent necrosis and inflammatory infiltrates observed in areas with hyperintense signal in the MRI, could be more related to the increased muscle T2 in the three dystrophic mouse strains, than the overall phenotype.

The T2 determination based on two echoes at two different TEs has long been dismissed as inadequate and imprecise. This needs to be revised and our data bring more evidence pointing into that direction. When image signal-to-noise ratio is high, two-point determination is accurate, often more than CPMG echo trains. In practice, scanner imperfections but also RF propagation through tissues create B1 field deviations that are responsible for the generation of stimulated echoes that bias the T2 decay. Most often, the measured mono-exponential T2 with this sequence is longer than the true T2. The single echo collection as was performed here prevents the introduction of stimulated echos. While obsolete at first glance, the method used here is in reality as valid as and probably more accurate than more popular methods.

We are here dealing with mono-exponential T2, that is classically used to characterize global water T2 dynamics in a given tissue. Muscle T2 decay is in reality multi-exponential and can be exploited to determine tissue water compartments and exchanges, as shown by the work by Saab [49] and more recently by Araujo [50]. It requires different and very demanding experimental approaches, which are seldom used in practice. In that respect, standard multi-echo sequences do not allow better than the 2 echoes method to tackle this multi-compartment organization of tissue.

In the comparison between MRI and the histological analysis, appropriate co-localization of the distribution of alterations was observed in the three dystrophic mouse strains, which is essential to validate the use of MRI as an outcome measure. In humans, a correlation between the degree of muscle involvement in MRI and the progression of dystrophic alterations in histological analysis was observed in DMD patients, but with MRI and muscle biopsy done in different muscles [51]. In the *mdx* mouse, our data comparing the histological analysis with the MRI in the same muscles showed that high intensity areas in muscle MRI were co-localized with variable dystrophic characteristics in the histological analysis, in accordance with the previous studies in this model [28]. Additionally, we extended for the first time this characterization for *Large* ^*myd*^ and *mdx/Large* ^*myd*^ dystrophic murine models, which showed a similar co-localization of alterations in the MRI versus the histological analysis. In all these dystrophic models the areas with increased muscle T2 in the MRI correlated spatially with several different histopathological alterations such as necrosis, inflammation, degeneration or regeneration foci. Thus, even if the increased muscle T2 could not differentiate individually each one of these pathological findings, the T2 maps reveal a disease process at a tissue level. Apart of the non-invasive nature, this MRI approach allows the evaluation of the dystrophic process along several muscles in the same exam, which is methodologically difficult considering the use of standard histological techniques.

Muscle T2 values were not correlated with the severity of the phenotype in the 3 dystrophic mouse strains, since the severely affected *Large* ^*myd*^ mice showed similar values than both the mild *mdx* and severe *mdx/Large* ^*myd*^ lineages. Notably, the major histological difference between these strains is fibrosis, which was not detectable with the MRI sequences used in this study. Connective tissue presents very short T2 values, being visualized only with appropriated pulse sequences such as Ultra Short Echo-time sequences (UTE) [52], which could not be used due to technical limitations. On the other hand, the MRI images showed clear different patterns of muscle signals. The muscle MRI was patchy in *mdx* mice, with delimited regions of increased MR signal distributed through the posterior limb muscles, both in the lower leg and in the calf images. The *Large* ^*myd*^ mice, by its turn, showed a waxy pattern in the muscle MRI, with a general increase in the MR signal distributed homogeneously through the lower leg and thigh muscles. Finally, the *mdx/Large* ^*myd*^ mice showed generally a diffuse increase in the MR signal, like the *Large* ^*myd*^ mice, but there were also hyperintense regions in the lower leg muscles, similar to what was observed in the *mdx* mice. After refining the muscle texture analysis with the evaluation of the co-occurrence matrix parameters entropy and contrast, it was possible to cluster the mice from different groups.

Changes in the muscle texture in small animals have already been reported in rats when comparing atrophic, regenerating and normal muscles [34]. In the *mdx* model, a heterogeneous muscle signal was also observed in a variable degree according mouse’s age, with a peak between the age of 5 and 17 weeks [36]. Additionally, texture parameters have been considered reliable biomarkers of disease progression in the GRMD dystrophic dog [53, 37]. Here we show the possibility of classifying murine models of muscle dystrophies with different phenotypes using quantitative NMR and texture analysis with prior knowledge. It is possible that the differences observed with our texture analysis were more evident due to the mice’s age, since it was close to the described peak of muscle heterogeneity in the *mdx* model [36]. Even though, these results indicate that MRI when combined to texture analysis can provide a refined noninvasive identification of muscle alterations, not only between drastically different conditions but also when more subtle differences are present. This approach can be potentially transferred to human applications. In this case, a preliminary step would be to select the most discriminant combination of texture indices in muscle MRI from patients with known diagnosis. Then, the model could be validated by investigating in which category new patients would be classified in confront with genetic analysis.

## Conclusion

While muscle mean T2 values were abnormal in all dystrophic muscle, and reflected the spatial distribution of different histopathological changes, they did not clearly distinguish the three different genotypes, nor were correlated to severity of the phenotype. On the other hand, texture analysis algorithms unambiguously separated muscles from *mdx, Large* ^*myd*^ and *mdx/Large* ^*myd*^ mice, reflecting the waxy versus patchy distribution of lesions in the different strains. Combined T2 maps and texture analysis provide a powerful non-invasive characterization of dystrophic muscles, even when performed at 2 teslas and derived from two single TEs measurements. Our findings have important implications to validate the use of MRI as an outcome measure in therapeutic protocols applied to mouse models of muscle dystrophies, with the possibility of direct applications in human translational research.

## Supporting Information

S1 Supporting Information T2 calculation from two images acquired at different echo times. Derivation of the calculation used to estimate T2 values from two images acquired at different echo times.
(DOC)

S2 Supporting Information Features selected for Texture Analysis. List of the 30 more relevant features automatically selected by the software MaZda 4.6 (F+PA+MI) for texture analysis, after manual selection of the co-occurrence matrix parameters contrast and entropy.
(DOC)
