# Supplementary material for: Quantitative T2 Combined with Texture Analysis of Nuclear Magnetic Resonance Images Identify Different Degrees of Muscle Involvement in Three Mouse Models of Muscle Dystrophy: mdx, Largemyd and mdx/Largemyd
Source: PLoS One. 2015 Feb 24;10(2):e0117835. doi: 10.1371/journal.pone.0117835 (PMC4339395; doi:10.1371/journal.pone.0117835)
Supplement: S1 Supporting Information — Derivation of the calculation used to estimate T2 values from two images acquired at different echo times. (DOC) [file pone.0117835.s001.doc]

**Supporting Information S1**

**T2 calculation from two images at different echo times**

The Bloch Equation for the spin-spin relaxation time (Equation 1) express the exponential decay of the sample magnetization as a function of the initial magnetization (M_0_), the echo time (TE), and the spin-spin relaxation time of the sample (T2):

(1) $M=M_{0}\exp\left(-\frac{TE}{T2}\right)$

If two images are collected at different echo times, TE_1_ and TE_2_, the ratio M_1_/M_2_ is given by:

(2) $\frac{M_{1}}{M_{2}}=\exp\left(-\frac{TE_{1}-TE_{2}}{T2}\right)$

The signal to noise ratio (SNR), corrected by the number of excitations used to compose the image (NEX), is proportional to the sample magnetization :

(3) $M\propto\frac{Signal}{Noise\sqrt{NEX}}$

Combining The Equations 2 and 3, the T2 value can be estimated by (4):

(4) $T2=\frac{\Delta TE}{\ln\left(\frac{SNR_{1}}{SNR_{2}}\right)}$

The equation (4) was the basis of the MatLab routine designed to calculate the T2 maps. The noise was estimated by measuring the mean signal in a region of interest (ROI) placed in an empty area from the images.
