# Supplementary material for: Quantitative T2 Combined with Texture Analysis of Nuclear Magnetic Resonance Images Identify Different Degrees of Muscle Involvement in Three Mouse Models of Muscle Dystrophy: mdx, Largemyd and mdx/Largemyd
Source: PLoS One. 2015 Feb 24;10(2):e0117835. doi: 10.1371/journal.pone.0117835 (PMC4339395; doi:10.1371/journal.pone.0117835)
Supplement: S2 Supporting Information — List of the 30 more relevant features automatically selected by the software MaZda 4.6 (F+PA+MI) for texture analysis, after manual selection of the co-occurrence matrix parameters contrast and entropy. (DOC) [file pone.0117835.s002.doc]

**Supporting Information S2**

**Features selected for Texture Analysis**

Two parameters related to the co-occurrance matrix, contrast and entropy, were manually selected. The co-occurrence matrix is the second order histogram of the image, with the estimate of the joint probability pdT(i,j) of two pixels, at distance d along a direction T, to have particular values i and j. MaZda calculates the co-occurrence matrix for up to 5 pixels of distance, and considering 4 directions (0º, 45º, 90º and 135º). The co-occurence matrix parameters are therefore computed for (d,0), (0,d), (d,d) and (d,-d), where d points to the distance between two pixels, which can take integer values from 1 to 5.

In the sequence, 30 features were automatically selected by combining the maximization of the Fischer coefficients, the maximization of the mutual information between two selected features, the minimization of the classification error probability and minimization of the average correlation coefficients (F+PA+MI). The selected features are listed below, and were used as input to Linear Discriminant Analysis (LDA).

|  | Feature |  | Feature |
| --- | --- | --- | --- |
| 1 | S(0,4)Contrast | 16 | S(1,0)Contrast |
| 2 | S(4,0)Contrast | 17 | S(5,0)Contrast |
| 3 | S(2,0)Entropy | 18 | S(5,5)Entropy |
| 4 | S(2,-2)Entropy | 19 | S(5,5)Contrast |
| 5 | S(5,-5)Entropy | 20 | S(0,4)Entropy |
| 6 | S(3,-3)Entropy | 21 | S(0,3)Entropy |
| 7 | S(4,-4)Entropy | 22 | S(2,2)Entropy |
| 8 | S(3,0)Entropy | 23 | S(0,2)Entropy |
| 9 | S(0,3)Contrast | 24 | S(3,3)Entropy |
| 10 | S(4,0)Entropy | 25 | S(1,-1)Entropy |
| 11 | S(5,-5)Contrast | 26 | S(1,1)Entropy |
| 12 | S(4,4)Entropy | 27 | S(0,1)Entropy |
| 13 | S(4,4)Contrast | 28 | S(1,0)Entropy |
| 14 | S(5,0)Entropy | 29 | S(0,2)Contrast |
| 15 | S(0,5)Entropy | 30 | S(2,-2)Contrast |
